# Antidiabetic and Cosmeceutical Potential of Common Barbery (*Berberis vulgaris* L.) Root Bark Extracts Obtained by Optimization of ‘Green’ Ultrasound-Assisted Extraction

**DOI:** 10.3390/molecules24193613

**Published:** 2019-10-08

**Authors:** Marina Dulić, Petar Ciganović, Lovorka Vujić, Marijana Zovko Končić

**Affiliations:** 1 Department of Pharmacognosy, Faculty of Pharmacy and Biochemistry, University of Zagreb, Marulićev trg 20, 10 000 Zagreb, Croatia; marinad4@net.hr (M.D.); petar.ciganovic@me.com (P.C.); 2 Department of Food Chemistry, Faculty of Pharmacy and Biochemistry, University of Zagreb, Domagojeva 1, 10 000 Zagreb, Croatia; lvujic@pharma.hr

**Keywords:** berberine, *Berberis vulgaris*, green extraction, glycerol, response surface methodology

## Abstract

*Berberis vulgaris* is rich in berberine, an isoquinoline alkaloid, with antidiabetic activity, often used topically for skin-related problems. The aim of this work was to develop a “green” method for berberine extraction using mixtures of water with glycerol, a non-toxic, environmentally-friendly solvent. Response surface methodology based on Box–Behnken design was used to optimize the experimental conditions for ultrasound-assisted extraction of berberine and anti-radical components from *B. vulgaris* root bark. The independent variables were temperature (X_1_), glycerol concentration (X_2_), and ultrasound power (X_3_), while the responses were berberine concentration and DPPH radical scavenging activity of the extracts (RSA IC_50_). The response values of the extracts prepared at optimum conditions were (response, X_1_, X_2_, X_3_): berberine yield (145.5 μg/mL; 80 °C, 50%, 144 W) and RSA IC_50_ (58.88 μL/mL; 80 °C, 30%, 720 W). The observed values deviated from the predicted values by −3.45% and 6.42% for berberine and RSA IC_50_, respectively, thus indicating the validity of the selected models. The prepared extracts demonstrated antioxidant, anti-melanogenic, and anti-inflammatory activity, as well excellent α-glucosidase and α-amylase inhibitory activity. The displayed biological properties and lack of glycerol toxicity makes the prepared extracts suitable for direct inclusion into antidiabetic and dermatologic food supplements and topical products.

## 1. Introduction

*Berberis vulgaris* L., Berberidaceae, is a deciduous shrub with a long history of medicinal and nutritional use in Europe, Asia, and America. While its fruit is mostly used as food, the root bark and stems of *B. vulgaris* have medicinal properties due to berberine, an isoquinoline alkaloid, mostly present in these organs [1]. Berberine and *Berberis* species display many pharmacological effects, including antidiabetic, anti-inflammatory, antioxidant [2], antibacterial [3], and antifungal effects [4]. Berberine is used in food supplements and dermatologic products. It is most commonly taken by mouth for diabetes, high cholesterol, and high blood pressure, or applied directly to the skin to treat burns and canker sores [5]. Although it is not suitable for use by children or during pregnancy and breastfeeding, berberine is considered safe for short-term use by adults when taken by mouth or applied to the skin [5].

Metabolic syndrome and type 2 diabetes are ailments that affect over 30–40% of the population older than 65 [6]. They are characterized by insulin resistance, hyperglycemia, as well as by overproduction of reactive oxygen species and a constant state of enhanced oxidative stress [7]. High blood glucose concentration in diabetes may cause polyol and hexosamine pathways, advanced glycation end-product formation, activation of protein kinase C, mitochondrial dysfunction, and consequently reactive oxygen species (ROS) accumulation. This leads to cellular damage and the development of diabetic complications, such as neuropathy, nephropathy, and retinopathy, as well as liver damage [7,8]. Berberine is well known for its anti-diabetic effects. For example, berberine lowers blood-glucose concentrations in healthy and diabetic people, and improves insulin secretion in healthy individuals [9]. In addition to this, it may reduce fasting and postprandial blood glucose, food, and water intake, as well as enhance antidiabetic effects of other drugs such as canagliflozin [10].

In addition to anti-diabetic activity, berberine has many properties that may be utilized for the development of cosmeceutical products. The word “cosmeceutical” is a marketing term often used in lay language to denote a topical product that possesses both cosmetic and dermatologic characteristics. In addition to hydrating properties, such products may display other activities, such as antioxidant, anti-wrinkle, skin-whitening, and anti-inflammatory activity among others. Among cosmeceuticals, the products that are derived from natural sources such as plants, are in special demand, not only because of the consumers’ preferences for natural skin-care, but also because of their numerous beneficial effects on human skin [11]. The documented antioxidant [2] and anti-inflammatory activity of barberry extracts and berberine [12], as well as its notable anti-wrinkle properties [13] make them suitable for inclusion in cosmeceutical products.

In order to incorporate berberine and other bioactive plants principles into the final products they need to be extracted from crude plant material. Ultrasound-assisted extraction (UAE) is an inexpensive and simple extraction technique, appropriate for extraction in solid/liquid systems. UAE is characterized by relatively high reproducibility, short time of extraction, low solvent consumption, as well as low extraction temperature and energy input. Among the numerous factors that may influence the efficiency of UAE, solvent type selection has been recognized as the most important. An efficient UAE process should maximize the recovery of target compounds with minimal degradation, resulting in an extract with high biological activity. Ideally, this should be accomplished using “green” environmentally friendly technologies and low-cost raw materials and solvents [14]. Because of its wide availability and lack of toxicity, water is the most appropriate solvent for the extraction of medicinal plants’ bioactive principles. It is often combined with ethanol to make it suitable for the extraction of non-polar bioactive molecules from plant material. However, in spite of its natural origin, the use of ethanol is limited by its flammability and skin-irritability. Furthermore, internal use of ethanol is not appropriate for children and members of certain religions. One of the solvents that could effectively replace ethanol for preparation of cosmetic products and food supplements is glycerol, a non-toxic, biodegradable liquid manufactured from renewable sources [15]. These characteristics make the extracts prepared using glycerol appropriate for the direct application in the formulation of the desired product, without the need for solvent removal. Interestingly, as opposed to optimization of ethanolic extraction, glycerol use for extraction of natural products is still under-researched. Few examples include the use of glycerol for extraction of polyphenolic antioxidants from two *Artemisia* species [16], olive (*Olea europaea*) leaves [17], and rice bran [18].

Seemingly similar extraction procedures can significantly affect the yield and the composition of plant extracts. Response surface methodology (RSM) is a statistical model-based methodology that determines the relationship between the extraction condition and one or more studied responses, thus decreasing the required time and cost of the experiments [19]. The aim of this work was to perform a comprehensive investigation of the influence of the extraction variables: temperature, glycerol concentration, and ultrasonication power (USP) on berberine content and radical scavenging activity (RSA) of *B. vulgaris* extracts using RSM. An additional goal was to test the biological activity of the prepared extracts using selected assays. Even though berberine can exert numerous beneficial effects on the human organism, its low oral bioavailability greatly limits its clinical application [20]. However, during the topical or oral application, the bioactive ingredients of *B. vulgaris* extracts may come into direct contact with skin or digestive tract enzymes, respectively. Therefore, in this work, the biological activity of *B. vulgaris* extracts was tested, targeting the activities relevant to cosmeceutical and digestive tract-related anti-diabetic applications.

## 2. Results

### 2.1. Response Surface Analysis of Berberine and RSA

UAE was employed to prepare *B. vulgaris* root bark extracts with high berberine content, as well as low RSA IC_50_ value. The independent variables selected for optimization were extraction temperature, concentration of glycerol in water, and USP. The research was carried out according to a three-factor Box–Behnken design (BBD) (Table 1). Preliminary experiments showed that water/glycerol mixtures are more suitable for *B. vulgaris* extraction than either pure water or glycerol. Therefore, 10–90% solutions of glycerol in water were used for the extraction in this work. Other extraction parameters were selected according to the maximum and minimum specifications of the ultrasonication bath. 

Table 2 shows the process variables and experimental data of 17 runs. The amount of extracted berberine greatly differed among extracts (Table 2). For example, run 13 contained only 32.46 μg/mL of berberine while run 17 contained as much as 146.65 μg/mL of the alkaloid. The radical scavenging activity of the extracts, investigated using 2,2-diphenyl-1-picrylhydrazyl (DPPH) free radicals, also varied significantly. In the presented work, RSA IC_50_ values of *B. vulgaris* extracts ranged between 55.35 μL/mL and 262.95 μL/mL. Butylated hydroxyanisole (BHA), used as the positive control in this assay, presented an RSA IC_50_ of 5.13 ± 0.18 μg/mL. Even though the results are not directly comparable because of different units, this indicates rather modest radical scavenging abilities of the extracts.

By applying multiple regression analysis on the experimental data, it was found that the relationship between the response variables and the independent variables can best be expressed by the quadratic polynomial equations. In order to achieve a better fit and thus observe the influence of the extraction conditions on RSA more clearly, the data were transformed using negative power (Table 3).

**Figure 1:**
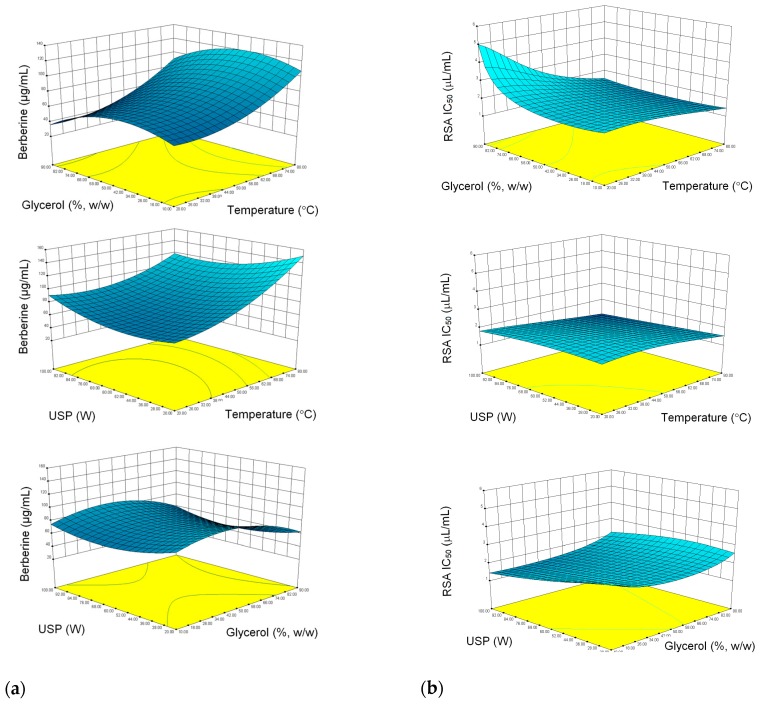
Response surface plots: influence of pairs independent variables on (**a**) berberine yield, and (**b**) radical scavenging activity (RSA IC_50_).

In order to enable visualization of the interactions between the independent and independent variables, the results are also presented as response surface plots. Figure 1 shows the surface plots of the influence of investigated UAE parameters on the berberine content (Figure 1a) and RSA IC_50_ (Figure 1b). From the plots and from Table 3, it is evident that the independent variables influenced the extraction in different manner. For example, temperature positively influenced the berberine concentration and RSA IC_50_^−1.78^ value both as linear and quadratic factors. This means that both berberine and the substances responsible for radical scavenging activity are better extracted at higher temperature. However, the combination of strong ultrasound and high temperature seemed to affect the berberine concentration negatively, while the effect on RSA IC_50_^−1.78^ remained positive. As evidenced by negative coefficients before quadratic and linear glycerol concentration, both berberine and substances with antiradical properties are better extracted using medium to low glycerol content. On the other hand, USP alone enhanced both berberine extraction efficiency (as quadratic factor) and RSA IC_50_^−1.78^ (as quadratic and linear factors). 

The analysis of variance (ANOVA) for the selected models (Table 4) has shown that the models are suitable for the description of the relationship of dependent and independent variables. The F-values of the models were higher than 26, while the *p*-values were lower than 0.0001. This indicates that both models are significant and that they were suitable for optimization of the extraction variables. Lack-of-fit test in both models was statistically insignificant relative to the pure error, meaning that the fitting model is adequate to describe the experimental data. The determination coefficients for both responses were relatively high (*r*^2^ > 0.97) showing good predictability of the results by the selected models. The adjusted and predicted *r*^2^ for both models were rather high and in good agreement. This further confirms the suitability of the models for the description of experimental data (Table 4). 

### 2.2. Optimization of Extraction Parameters and Model Validation

The aim of this study was to maximize the berberine extraction yield, as well as to minimize the RSA IC_50_ of the *B. vulgaris* extracts. In order to establish the optimum levels of the independent variables, numerical optimizations have been conducted based on the experimental results and the statistical analysis. Two extracts, one with maximized berberin content (B-opt) and the other with minimized RSA IC_50_ value (RSA-opt) were prepared. Optimal extraction conditions and the predicted values of corresponding responses are presented in Table 5. As expected from the polynomial equations, high temperature beneficially affected both berberine concentration and RSA. Somewhat higher glycerol content and lower USP was needed for optimal berberine yield in comparison to those needed for optimal RSA of the extracts.

Berberine content and RSA IC_50_ value were determined in both extracts. In addition to the values presented in Table 5, B-opt had an RSA IC_50_ value of 77.37 μL/mL, while the berberine content in RSA-opt was 116.9 μg/mL.

### 2.3. Antioxidant Activity of the Extracts

**Figure 2:**
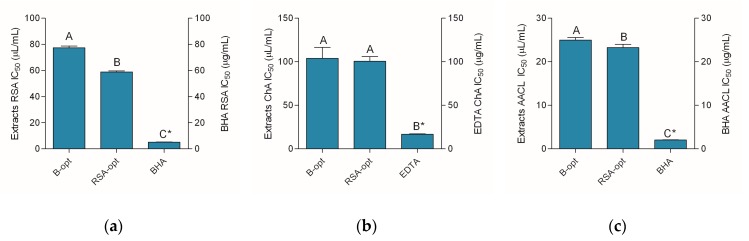
Antioxidant activity of the extracts and positive controls—BHA (butylated hydroxyanisole) and EDTA (ethylenediaminetetraacetic acid): (**a**) antiradical activity, (**b**) chelating activity, and (**c**) the activity in β-carotene-linoleic acid assay. Different uppercase letters indicate statistical significance (*p* < 0.05). Asterisks (*) indicate that the IC_50_ unit is placed on the right *y*-axis.

RSA, chelating activity on Fe^2+^ ions, and the activity in heat-induced degradation of β-carotene-linoleic acid system were investigated (Figure 2). It is important to note that the activity of the extracts may not be directly compared to the standard antioxidants due to the fact that the activity was expressed in different measurements units (the activity of the extracts and standards were expressed as μL/mL and μg/mL, respectively). However, for comparison purposes, it is possible to regard the activity of the standards as volume equivalents of 1 mg/mL solutions.

The data presented in Figure 2 indicate mild to moderate antioxidant activity of the extracts in comparison to the standards. Expectedly, RSA-opt was a stronger radical scavenger, while a somewhat lower level of scavenging activity is demonstrated by B-opt (Figure 2a). Similarly, RSA-opt was better capable of hindering oxidation of linoleic acid in a β-carotene-linoleic acid assay (Figure 2c), while the capability of the extracts to chelate Fe^2+^ ions was statistically equal (Figure 2b). 

### 2.4. Tyrosinase-, Lipoxygenase-, and Coagulation-Inhibiting Activity

**Figure 3:**
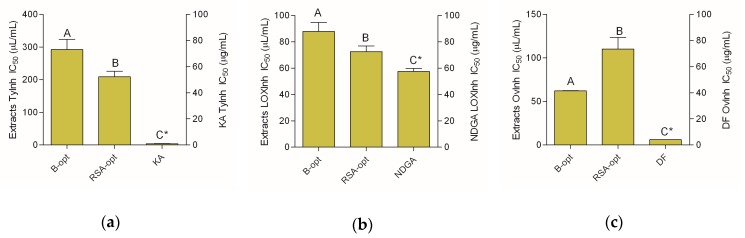
Cosmeceutical activity of the extracts and positive controls—kojic acid (KA), nordihydroguaiaretic acid (NDGA), and diclofenac (DF): (**a**) tyrosinase-inhibiting activity, (**b**) lipoxygenase (LOX)-inhibiting activity, and (**c**) coagulation-inhibiting activity. Different uppercase letters indicate statistical significance (*p* < 0.05). Asterisks (*) indicate that the IC_50_ unit is placed on the right *y*-axis.

The cosmeceutical potential of the prepared extracts was investigated by studying their tyrosinase- and lipoxygenase (LOX)-inhibiting properties. Furthermore, the ability to inhibit heat-induced protein coagulation was also investigated. Both extracts were active in the performed assays but to varying degrees (Figure 3).

Although the extracts displayed some level of anti-tyrosinase activity, their activity was rather low in comparison to kojic acid, the standard skin-whitening substance (Figure 3a). It may also be noted that the RSA-opt was significantly more active than B-opt in this assay. Furthermore, RSA-opt was a stronger LOX inhibitor than B-opt. Its activity was relatively close to the activity of 1 mg/mL nordihydroguaiaretic acid (NDGA) solution (Figure 3b). Even though both extracts were weaker inhibitors of heat-induced protein coagulation compared with diclofenac, B-opt displayed stronger activity than RSA-opt in this assay (Figure 3a).

### 2.5. α-Glucosidase- and α-Amylase-Inhibiting Activity

**Figure 4:**
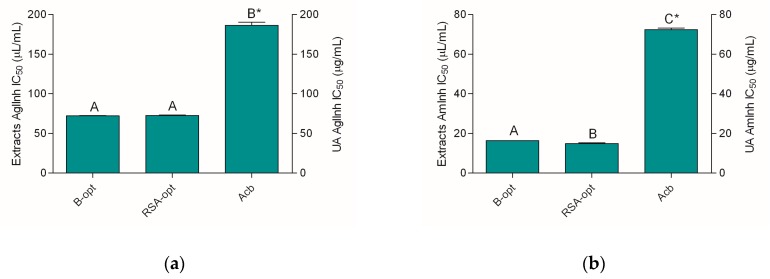
Antidiabetic activity of the extracts and positive control acarbose (Acb): (**a**) α-glucosidase-inhibiting activity and (**b**) α-amylase-inhibiting activity. Different uppercase letters indicate statistical significance (*p* < 0.05). Asterisks (*) indicate that the IC_50_ unit is placed on the right *y*-axis.

The anti-diabetic potential of the extracts was investigated by studying their potential to inhibit two enzymes involved in carbohydrate digestion: α-glucosidase and α-amylase. The extracts displayed a similar and rather notable inhibition of these two enzymes (Figure 4).

The tested extracts were equally able to impair α-glucosidase activity. Interestingly, their activity was even higher than the activity of 1 mg/mL acarbose solution (Figure 4a). Similarly, the extracts were rather strong α-amylase inhibitors. The IC_50_ values of the extracts amounted to approximately one-half and one-third of the IC_50_ value of the acarbose solution in α-glucosidase and α-amylase assays, respectively. Although the activities of the extracts were rather similar, RSA-opt displayed slightly better inhibiting properties in the α-amylase assay.

## 3. Discussion

### 3.1. Response Surface Analysis and Optimization of Extraction Parameters

*Berberis vulgaris* root bark is a rich source of berberine, an isoquinoline alkaloid, with various beneficial health-related properties [3]. In this work, a “green” UAE extraction of bioactive principles from *B. vulgaris* root bark was optimized using RSM. Since this work was directed at the preparation of extracts suitable for direct use in cosmeceutical products and anti-diabetic food supplements, mixtures of water with glycerol, a non-toxic and environmentally friendly liquid of natural origin with humectant properties and a very low glycemic index, was chosen as the extraction solvent. 

The extraction conditions for bioactive natural products must be carefully selected to ensure that the prepared extracts have the desired characteristics [21]. In order to achieve this, various RSM models are used. In this work, BBD was successfully applied for the optimization of berberine and antiradical compound extraction from *B. vulgaris* root bark. The first of the two optimized parameters, berberine concentration, was chosen because berberine is the most important and well-known constituent of *B. vulgaris* root. Most of the observed bioactivity of this herbal drug is attributed specifically to its berberine content [2,5]. The other dependent variable, the antioxidant activity of the extracts, was selected because it is important for both potential antidiabetic and cosmeceutical activity, as will be discussed later. Various phytochemicals present in *B. vulgaris* and other medicinal plants may display antioxidant activity. When investigating extraction with solvents of changing polarity, it is possible that one group of substances is the main responsible for the activity in predominantly hydrophilic extracts and the other in hydrophobic extracts. Therefore, the activity is not necessarily directly correlated either with the concentration of one antioxidant or even the sum of all antioxidants present in the solution (e.g., in case of synergism). Thus, instead of quantifying various phytochemicals, the target activity itself was determined and optimized. Among the many assays described in the literature, DPPH assay was selected as a simple, reliable and straightforward test, suitable for determination of antiradical activity of a large number of natural extracts in relatively short time. In spite of some limitations, these characteristics render the DPPH assay one of the most commonly used models for the determination of antioxidant activity in the scientific literature today [22].

While UAE may greatly improve the yield of the extraction in comparison to classical techniques such as maceration, it may also affect the composition and the biological activity of the prepared extracts, especially if target compounds are sensitive to degradation. It has been previously noted that the UAE extraction conditions strongly influence the biological activity of the extracts from different *Berberis* sp., such as in the antitumor activity of *B. amurensis* extracts [23].

In this work, high temperature positively affected the berberine content of the extracts. Similar to this, USP also positively influenced berberine concentration, while the influence of glycerol concentration was predominantly negative. While this is the first detailed analysis of the USP effect on berberine concentration, the positive influence of high temperature was previously recorded for ethanolic UAE of Rhizoma Coptidis [24]. High temperature and USP may improve the extraction process by reducing the viscosity of the solvent and increasing the kinetic energy of the molecules in the solutions. In addition to this, higher USP induces more damage to cell walls, thus releasing more intracellular components which can then partition into the extracting solvent [25]. It is interesting to note that, in this work, the combination of high USP and high temperature acted detrimentally on berberine concentration. The observed reduction of berberine concentration may be attributed to its degradation caused by hydroxyl radicals [26] whose production is initiated by ultrasonication, especially at high temperatures [27].

The negative influence of glycerol concentration on extraction of antiradical principles of *B. vulgaris* indicates relatively high polarity of the substances responsible for radical scavenging effects of the extracts. Temperature, USP, and their interaction, on the other hand, positively affected RSA of the extracts. Even though there are no specific studies investigating influence of those parameters on RSA of *B. vulgaris*, some reports show that low USP [28] or temperature [29] are beneficial for DPPH antiradical activity of plant extracts, while others demonstrate that the moderate-to-high temperature [30] or USP [31] may beneficially affect RSA. This is not surprising, as numerous plant components with different physicochemical properties may display RSA, and extraction conditions are do not influence all of them in the same manner. The overall positive influence of temperature and USP on RSA found in this work thus indicates good thermal and chemical stability of the radical scavengers present in the extracts. It is important to note that, even though berberine shows some degree of antiradical activity [32], the RSA IC_50_ of the extracts in this work was not in correlation with berberine content. This means that, besides berberine, other phytochemicals also contribute to the observed antiradical effects, as discussed below.

### 3.2. Antioxidant Activity of the Extracts

Antioxidant activity is a very important characteristic for the proper storage and function of cosmeticeutical products and anti-diabetic food supplements. Antioxidants may protect the product against the oxidation that occurs during its storage and use. Free radical or metal ions may induce peroxidation of polyunsaturated fatty acids in liquid and semi-solid dosage forms, thus impacting not only the quality but also the safety of the product [33]. In addition to this, antioxidant functional ingredients may have a more active role in such products. For example, antioxidants in topical products may offer protection against oxidative damage of skin macromolecules associated with the effects of free radicals and UV radiation [34]. Antioxidants have a very important role in the prevention and treatment of type 2 diabetes. It is well known that chronic exposure to high glucose concentration, as is the case in diabetes, depletes the levels of endogenous antioxidants and produces oxidative stress in various tissues. This may trigger irreversible damage of the affected cells, ultimately leading to apoptosis. Natural metabolites and extracts may prevent oxidative changes, normalize the concentration of intracellular antioxidants, and thus prevent or even reverse cell damage in vivo and in vitro [35]. The relatively modest antioxidant activity of the extracts observed in this work was not surprising. Some studies have demonstrated good RSA, chelating activities, and other types of antioxidant activity of *B. vulgaris* fruit [36] and leaves [37,38]. However, the activity of *B. vulgaris* root, although measurable, was always significantly lower than the activity of controls, such as ascorbic acid [39,40]. Previous studies have shown that the phenolics substances [37] (e.g., cannabisin G and (±)-lyoniresinol [41]) are the main substances responsible for RSA activity of the *B. vulgaris* root bark extracts. Furthermore, it was found that polysaccharides are the important radical scavengers in *B. dasystachya* [42]. In addition to this, berberine also displays some degree of DPPH radical scavenging and iron chelating activity. However the same work also reports that the activity in both assays was much lower than the activity of the used standard, ascorbic acid [32]. Although determining the exact structures and quantities of the substances responsible for the observed in vitro activity is outside of scope of this research, it was most likely that numerous antiradical compounds contributed to the observed RSA. This may have included berberine together with other phytochemicals present in the root bark, such as various phenolics and polysaccharides. Although the antioxidant activity of the extracts in the performed assays was rather modest, it still positively contributes to their potential use in various food supplements and cosmeceutical products.

### 3.3. Tyrosinase-, Lipoxygenase-, and Coagulation-Inhibiting Activity

Besides simple hydration and antioxidant protection, cosmeceutical products should also display other biological properties beneficial for skin. Previous research has shown that berberine and the plants and formulations that contain berberine may have an anti-inflammatory effect. Berberine can suppress the release of interleukins in the eosinophil culture and decrease the expression of tyrosinase [43]. In order to further assess the cosmeceutical potential of the prepared extracts, anti-tyrosinase and anti-LOX, as well as anti-inflammatory activity against protein coagulation, were investigated. Tyrosinase inhibitors block melanogenesis by inhibiting tyrosine oxidation to dopaquinone, thus preventing hyperpigmentation of the skin. They are used in treatment of skin discolorations, such as in the treatment of melasma or lentigo solaris [44]. Anti-inflammatory activity is also important for cosmeceutical products. Skin inflammation can be defined as the skin response to an injury, infection, or destruction. It is usually characterized by heat, redness, pain, swelling, or disturbed skin physiological functions. Many dermatologic diseases, such as atopic dermatitis or acne vulgaris, are characterized by inflammatory processes [45]. In this work, anti-inflammatory potential of the extracts was investigated using two assays. The first was the LOX-inhibition assay. LOX is the enzyme involved in arachidonic acid metabolism and the release of various pro-inflammatory eicosanoid substances, such as leukotrienes and lipoxins. LOX plays an important role in the elicitation of skin inflammation and mediates the inflammatory events that are developed as a result of various environmental factors, such as ultraviolet radiation, inflammation mediators, and allergens [46]. The second assay was the inhibition of protein coagulation. Denaturation of tissue proteins is one of the characteristics that causes inflammatory processes. Therefore, the suppression of protein denaturation hinders the development of inflammation-related skin changes, which is another important aspect of anti-aging activity [47]. While the direct tyrosinase-inhibiting and anti-inflammatory activity of *B. vulgaris* was not investigated before, the standardized *B. aristata* extracts were mixed-type tyrosinase inhibitors [48]. In addition to this, berberine from the antipsoriatic plant *Mahonia aquifolium* displayed only a very weak anti-LOX activity [49]. The extracts prepared in this study were active in all the applied assays. Their ability to inhibit the melanogenesis and inflammatory changes caused by LOX activity and protein coagulation makes the extracts potentially good candidates for inclusion into cosmeceutical products. 

### 3.4. α-Glucosidase- and α-Amylase-Inhibiting Activity

Antidiabetic activity of plant extracts may be related to their influence on the enzymes that participate in polysaccharide digestion, thus impairing their degradation to glucose and other monosaccharides. The enzyme α-amylase is secreted in saliva and pancreatic juice. It catalyzes the hydrolysis of starch to a mixture of smaller oligosaccharides, which are then degraded to glucose by α-glucosidase, an enzyme located in the mucosal brush border of the small intestine. Therefore α-amylase and α-glucosidase inhibitors of natural origin can be of importance in the development of drug leads intended for the treatment of diabetes, obesity, and hyperlipemia. In accordance with some previous findings obtained using ethanolic [50] and methanolic [51] *B. vulgaris* extracts, the extracts used in this study demonstrated excellent inhibition of α-glucosidase and α-amylase. An increasing number of studies have shown that berberine significantly accumulates in the intestines [52]. Therefore α-glucosidase and α-amylase inhibitory activity of glycerol and other *B. vulgaris* extracts can certainly contribute to well-established antidiabetic properties of berberine and plants that contain it.

## 4. Materials and Methods 

### 4.1. Plant Materials and Chemicals

Root bark of *Berberis vulgaris* was a gift from Suban (Samobor, Croatia). Berberine chloride (≥98.5%), BHA (≥98.5%), kojic acid, diclofenac, α-glucosidase, α-amylase, LOX, and tyrosinase were purchased from Sigma-Aldrich (St.Louis, MO, USA), while soybean LOX was from purchased from TCI chemicals (Tokyo, Japan). Acetonitrile was of HPLC grade. Other reagents and chemicals were of analytical grade. 

### 4.2. Preparation of Extracts

Prior to the extraction, plant material was grinded and passed through a sieve of 850 μm mesh size. Powdered plant material (0.2 g) was suspended with 10 mL of the appropriate solvent in a 50 mL Erlenmeyer flask. The extraction was performed in an ultrasonic bath (Bandelin SONOREX Digital 10 P DK 156 BP, Berlin, Germany) at 35 Hz suitable USP. The bath was temperature-controlled. The extraction conditions were chosen according to the Box–Behnken design (Table 1 and Table 2). Upon the extraction, the mixture was filtered using folded filter papers S&S 589/1 1/2 (Schleicher & Schuell, Keene, NH, USA). All the extracts were stored at +4 °C in the dark until use.

### 4.3. Experimental Design

Design Expert software version 8.0.6 (Stat-Ease, Minneapolis, MN, USA) was employed for the regression analysis and the optimization of the results. A three-level-three-factor BBD was employed to determine the best combination of independent extraction variables for the selected dependent variables. The coded values for design parameters (dependent variables) were chosen as presented in Table 1. Berberine concentration (Y_1_) and RSA IC_50_ (Y_2_) were selected as the responses (Table 2). Experimental data were fitted to a quadratic polynomial model, as described by the quadratic Equation (1):

(1) $$Y=A_{0}+\overset{k}{\underset{i=1}{\sum }}A_{i}X_{i}\;+\overset{k}{\underset{i=1}{\sum }}A_{\mathrm{ii}}X_{i}^{2}+\overset{k-1}{\underset{i=1}{\sum }}\times \overset{k}{\underset{j=1+1}{\sum }}A_{\mathrm{ij}}X_{i}X_{j}$$

where Y is the dependent variable; A_0_, A_i_, A_ii_, and A_ij_ are the regression coefficients for the intercept, linearity, square, and interaction, respectively; X_i_ and X_j_ are the independent variables.

### 4.4. Berberine Quantification

Berberine was quantified using an HPLC instrument (Agilent 1200 series, Agilent Technologies, USA) equipped with a diode array detector (DAD) and Zorbax Eclipse XDB C18 column (5 μm, 250 mm × 4.6 mm, Agilent Technologies, Santa Clara, CA, USA). The injection volume was 20 μL. Before the injections, the solutions of the standard (0.2 mg/mL solution of berberine) and the extracts were filtered through a 0.45 μm PTFE-syringe filter. Triethylamine-adjusted 0.02 mol/L H_3_PO_4_ (pH 4.82) with 25% acetonitrile was chosen as the mobile phase. The flow-rate was 1.0 mL/min. The peaks were observed and quantified at 254 nm. The peak assignment and identification was based on a comparison of retention times and the spectra of peaks in the sample chromatogram with those of the standard. Berberine was quantified according to its respective standard calibration curve. The calibration curve was plotted as area under curve (AUC) of berberine peak (*y*, arbitrary units) against the weight of berberine in the sample (*x*, μg). Limit of detection (LD) and limit of quantification (LQ) were determined according to [53]. LD and LQ were 0.0186 μg, 0.0564 μg, respectively, while the calibration curve is presented in Equation (2):

y = 2834.4x + 22.08 (*r*^2^ = 0.9999) (2)

### 4.5. Free Radical Scavenging Activity

RSA was evaluated as described by Fumić et al. [30]. Methanolic solution of DPPH (70 μL, 0.21 mg/mL) was added to 130 μL of either the methanolic solution of the extract (sample) or methanol (negative control). After 30 min in the dark at room temperature, the absorbance was read at 545 nm using microplate reader (Stat Fax 3200, Awareness Technologies, Palm City, FL, USA). RSA was calculated according to Equation (3):

(3) $$\mathrm{RSA}\;\left(\%\right)=\frac{A_{control}-A_{sample}}{A_{control}}\times 100$$

where *A_control_* is the absorbance of the negative control and *A_sample_* is the absorbance of the DPPH solution containing extract. Concentration of the extract, which scavenged 50% of DPPH free radicals present in the solution (RSA IC_50_), was calculated and expressed as μL of extract/mL of solution (μL/mL). BHA was used as the standard radical scavenger.

### 4.6. Fe^2+^ Chelating Activity

The chelating activity (ChA) of the investigated substances toward ferrous ions was studied, as described by Bljajić et al. [8]. To the solution of the extract in methanol (150 μL), 0.25 mM of FeCl_2_ solution (50 μL) was added. After 5 min, 100 μL of 1.0 mM ferrozine solution was applied. Absorbance at 545 nm was recorded after 10 min. Reaction mixture containing methanol (150 μL), instead of the extract, served as a negative control. ChA was calculated using Equation (4):

(4) $$ChA\;\left(\%\right)=\frac{A_{control}-A_{sample}}{A_{control}}\times 100$$

where *A_control_* is the absorbance of the negative control and *A_sample_* is the absorbance of the respective extract. Concentration of the extract, which chelated 50% of Fe^2+^ present in the solution (ChA IC_50_), was calculated and expressed as μL of extract/mL of solution (μL/mL). Ethylenediaminetetraacetic acid (EDTA) was used as the chelating standard.

### 4.7. Antioxidant Activity in β-Carotene-Linoleic Acid Assay

Antioxidant activity in β-carotene-linoleic acid assay (AOA) was evaluated using the β-carotene-linoleic acid system according to modified literature procedure published by Rajić et al. [54]. Aliquots (200 μL) of the emulsion containing β-carotene (6.7 μg/mL), linoleic acid (0.7 mg/mL), and Tween 40 (6.7 mg/mL) were added either to water (50 μL) (control) or to the solutions of the extract in methanol (50 μL). The reaction mixture was incubated at 50 °C. The antioxidant activity in β-carotene linoleic acid assay (AACL) was calculated based on the absorbance recorded after 60 min using Equation (5):

(5) $$AACL\;\left(\%\right)=\frac{A_{sample}}{A_{control}}\times 100$$

where *A_control_* and *A_sample_* are the absorbances of the water control and the antioxidant, respectively. Concentration of the extract, which protected 50% β-carotene present in the solution (*AACL* IC_50_), was calculated and expressed as μL of extract/mL of solution (μL/mL). BHA was used as the standard antioxidant.

### 4.8. Tyrosinase Inhibitory Activity 

Tyrosinase inhibition activity of the extracts was determined following the method described by Masuda et al. [55] with minor modifications. In 80 μL extract solution or water (negative control), 40 μL of mushroom tyrosinase solution (in 16 mM, pH 6, 8 phosphate buffer) was added. The solution was incubated in the dark at 25 °C. After 10 min, 80 μL of 3,4-dihydroxy-L-phenylalanine (L-DOPA) solution (0.19 mg/mL in phosphate buffer) was added. After the additional 10 min, the absorbance at 492 nm was measured. The negative control contained buffer instead of the extract solution. Tyrosinase inhibitory activity (TyInh) was calculated as described in Equation (6):

(6) $$TyInh\;\left(\%\right)=\frac{A_{control}-A_{sample}}{A_{control}}\times 100$$

where *A_control_* is the absorbance of the negative control and *A_sample_* is the absorbance of the respective extract. Concentration of the extract, which inhibited 50% of tyrosinase activity (*TyInh* IC_50_), was calculated and expressed as μL of extract/mL of solution (μL/mL). Kojic acid was used as the standard inhibitor.

### 4.9. Lipoxygenase Inhibitory Activity 

LOX inhibitory activity was determined spectrophotometrically [56]. A volume of 50 µL of different concentrations of extracts or water (negative control) was mixed with 150 μL phosphate buffer (pH 8, 100 μM) and 20 μL of soybean LOX solution. Finally, 30 μL of linoleic acid was added to initiate a reaction. The mixtureas incubated at 25 °C for 10 min and the absorbance was determined at 234 nm. LOX inhibitory activity (LOXInh) was calculated as presented in Equation (7):

(7) $$LOXInh\;\left(\%\right)=\frac{A_{control}-A_{sample}}{A_{control}}\times 100$$

where *A_control_* is the absorbance of the negative control and *A_sample_* is the absorbance of the respective extract. Concentration of the extract, which inhibited 50% of LOX activity (*LOXInh* IC_50_), was calculated and expressed as μL of extract/mL of solution (μL/mL). Nordihydroguaiaretic acid (NDGA) was used as a positive control. 

### 4.10. Anti-Inflammatory Activity 

Anti-inflammatory activity was evaluated using the heat-induced ovalbumin coagulation method [47]. The reaction mixture consisted of 0.4 mL of ovalbumin solution (50% fresh hen’s albumen), 2.8 mL of phosphate buffered saline (pH 6.4), and 2 mL of the extract solution or water (negative control). The mixtures were incubated at 37 °C for 15 min and then heated at 70 °C for 5 min. After cooling, their absorbance was recorded at 660 nm. The percentage inhibition of ovalbumin denaturation (OvInh) was calculated by using the following Equation (8):

(8) $$OvInh\;\left(\%\right)=\frac{A_{control}-A_{sample}}{A_{control}}\times 100$$

where *A_control_* is the absorbance of the negative control and *A_sample_* is the absorbance of the respective extract. Concentration of the extract, which inhibits 50% of the ovalbumin coagulation (*OvInh* IC_50_), was calculated and expressed as μL of extract/mL of solution (μL/mL). Diclofenac sodium was used as the standard inhibitor.

### 4.11. α-Glucosidase Inhibition Assay

Inhibition of *α*-glucosidase was determined spectrophotometrically [8] with slight modification. In brief, 20 μL of test samples or water (negative control) were incubated with 50 μL of *α*-glucosidase from *Saccharomyces cerevisiae* (0.2 U/mL dissolved in 0.1 M phosphate buffer, pH 6.8) for 10 min at 37 °C. Afterwards, 50 μL substrate (1 mM *p*-nitrophenyl-*α*-D-glucopyranoside prepared in same buffer) was added to the reaction mixture and the release of *p*-nitrophenol was measured at 405 nm after 5 min of incubation. Percentage of *α*-glucosidase inhibition (AglInh) was calculated as follows, according to Equation (9):

(9) $$AglInh\;\left(\%\right)=\frac{A_{control}-A_{sample}}{A_{control}}\times 100$$

where *A_control_* is the absorbance of the negative control and *A_sample_* is the absorbance of the reaction mixture containing extracts. Concentration of the extract, which inhibited 50% *α*-glucosidase activity (*AglInh* IC_50_), was calculated and expressed as μL of extract/mL of solution (μL/mL). Standard reference acarbose was used.

### 4.12. α-Amylase Inhibition Assay

The assay was performed according to Apostolidis et al. [57]. Extracts (0.5 mL) at different concentrations, or water (negative control), and 0.5 mL of 20 mM phosphate buffer (pH 6.9) containing α-amylase from porcine pancreas (0.8 U/mL) were preincubated at 25 °C for 10 min. This was followed by the addition of 0.5 mL soluble starch (0.5% solution in the same buffer). The reaction mixtures were incubated at 25 °C for 10 min and then the reaction was stopped with 1 mL of 96 mM 3.5-dinitrosalicylic acid color reagent. Afterwards, the test tubes were incubated in a boiling water bath for 5 min and cooled to room temperature. The reaction mixtures were diluted by adding 10 mL distilled water and absorbance was measured at 540 nm, and percentage of α-amylase inhibition (AmInh) was calculated, as shown in Equation (10):

(10) $$AmInh\;\left(\%\right)=\frac{A_{control}-A_{sample}}{A_{control}}\times 100$$

where *A_control_* is the absorbance of the negative control and *A_sample_* is the absorbance of the reaction mixture containing extracts. Concentration of the extract, which inhibited 50% amylase activity (*AmInh* IC_50_), was calculated and expressed as μL of extract/mL of solution (μL/mL). Acarbose was used as the positive control.

### 4.13. Statistical Analysis

The measurements were performed in triplicate and the results are presented as mean ± standard deviation. In order to establish the IC_50_ values, the experiments were performed using different concentrations (four to seven concentrations, depending on the assay). Statistical comparisons were made using one-way ANOVA, followed by Tukey’s post-hoc test for multiple comparisons (GraphPad Prism, San Diego, CA, USA). *p* < 0.05 was considered statistically significant. IC_50_ values were calculated by applying the appropriate regression analysis.

## 5. Conclusions

Glycerolic UAE procedure of berberine and antiradical components from *B. vulgaris* was developed using RSM. The prepared extracts were efficient radical scavengers and Fe^2+^ ion chelators. Furthermore, they were able to impair heat-induced degradation proteins and linoleic acid and. In addition to this, the prepared extracts were efficient tyrosinase, LOX, α-glucosidase, and α-amylase inhibitors. Because of their excellent cosmeceutical and anti-diabetic properties, as well as the non-toxicity of the solvent used for the extraction, the prepared extracts are suitable candidates for direct use in antidiabetic and dermatologic food supplements and topical products.

## Figures and Tables

**Table 1:** Independent variables and their levels for Box–Behnken design.

Independent Variables	Code	Levels		
		−1	0	1
Temperature °C	X1	20	50	80
Glycerol concentration (%, *w*/*w*)	X2	10	50	90
Ultrasonication power (USP) (W)	X3	144	432	720

**Table 2:** The Box–Behnken design and results of experiments.

Run	Standard	X_1_ (°C)	X_2_ (%, *w*/*w*)	X_3_ (W)	Berberine	RSA IC_50_
					μg/mL	μL/mL
1	1	20	10	432	57.11	108.73
2	15	50	50	432	75.06	103.47
3	9	50	10	144	84.58	134.14
4	16	50	50	432	69.00	90.84
5	14	50	50	432	83.33	83.79
6	4	80	90	432	89.79	74.37
7	10	50	90	144	68.53	128.59
8	12	50	90	720	59.98	113.51
9	5	20	50	144	69.27	98.35
10	17	50	50	432	84.28	83.63
11	2	80	10	432	111.03	72.98
12	13	50	50	432	72.56	86.46
13	3	20	90	432	32.46	262.95
14	8	80	50	720	120.8	55.35
15	7	20	50	720	95.58	87.74
16	11	50	10	720	69.62	72.59
17	6	80	50	144	146.65	78.86

X_1_ = temperature, X_2_ = glycerol content, X_3_ = ultrasonication power; RSA = radical scavenging activity.

**Table 3:** Polynomial equations of the models in terms of coded factors.

Response	Unit	The Equation Coefficients: *a* × X_1_^2^ + *b* × X_2_^2^ + *c* × X_3_^2^ + *d* × X_1_ × X_2_ + *e* × X_1_ × X_3_ + *f* × X_2_ × X_3_ + *g* × X_1_ + *h* × X_2_ + *i* × X_3_ + *j*									
		*a*	*b*	*c*	*d*	*e*	*f*	*g*	*h*	*i*	*j*
Berberine	(μg/mL)	16.57 *	−20.82 *	14.65 *	0.85	−13.04 *	1.60	26.73 *	−8.95 *	−2.88	76.85
RSA IC_50_^−1.78^	(mL/mL)	0.089 *	−0.12 *	0.039	0.045	0.081 *	−0.074 *	0.16 *	−0.061 *	0.11 *	0.36

X_1_ = temperature (°C), X_2_ = glycerol content (%, m/m), X_3_ = ultrasonication power (W). * = the significant equation terms (*p* < 0.05).

**Table 4:** Analysis of variance (ANOVA) for the fitted quadratic polynomial model for optimization of extraction parameters.

	Berberine *r*^2^ = 0.9720; *r*_adj_^2^ = 0.9359; *r*_pr_^2^ = 0.7850					RSA (IC_50_) *r*^2^ = 0.9740; *r*_adj_^2^ = 0.9406; *r*_pr_^2^ = 0.9034				
Source	SS	df	MS	*F*-Value	*p*-Value	SS	df	MS	*F*-Value	*p*-Value
Model	10834.44	9	1203.83	26.957	0.0001	4.35 × 10^−7^	9	4.84 × 10^−8^	29.17	<0.0001
Lack of fit	132.178	3	44.06	0.977	0.4870	1.74 × 10^−9^	3	5.79 × 10^10^	0.23	0.8683
Pure error	180.436	4	45.11			9.87 × 10^−9^	4	2.47 × 10^−9^		

*r*_adj_^2^ = adjusted *r*^2^; *r*_pr_^2^ = predicted *r*^2^; RSA = radical scavenging activity; SS = sum of squares; df = degrees of freedom; MS = mean square.

**Table 5:** Predicted and observed values for the optimized response variables.

Extract Name	Optimized Response	Aim of the Optimization	X_1_ °C	X_2_ %	X_3_ W	Predicted	Observed	RD (%)
B-opt	Berberine (μg/mL)	maximized	80	50	144	150.7	145.5	−3.45
RSA-opt	RSA IC_50_ (μL/mL)	minimized	80	30	720	55.33	58.88	6.42

X_1_ = temperature, X_2_ = glycerol content, X_3_ = ultrasonication power; RD = response deviation, calculated as (observed − predicted)/predicted × 100.
